# Are Presence/Absence Microbial Tests Appropriate for Monitoring Large Urban Water Supplies in Sub-Saharan Africa?

**DOI:** 10.3390/w11030491

**Published:** 2019-03-08

**Authors:** Clara MacLeod, Rachel Peletz, Francis Kere, Aminata M’ Baye, Michael Onyango, Sadat Aw, Mamadou El Hadj Issabre, Rosalind Tung, Ranjiv Khush

**Affiliations:** 1 Aquaya Institute, PO Box 21862-00505, Nairobi 00100, Kenya; 2 Aquaya Institute, PO Box 1603, San Anselmo, CA 94797, USA; rachel@aquaya.org (R.P.); ranjiv@aquaya.org (R.K.); 3 Office National de l’Eau et de l’Assainissement (ONEA), Ouagadougou 01, Burkina Faso; dfkere@yahoo.fr; 4 Sénégalaise des Eaux (SDE), Dakar 10200, Senegal; afmbaye@sde.sn; 5 Nairobi City Water and Sewerage Company (NCWSC), Nairobi 00100, Kenya; monyango@nairobiwater.co.ke; 6 Société de Distribution d’Eau de la Côte d’Ivoire (SODECI), Abidjan 01, Côte d’Ivoire; awsadat@sodeci.ci; 7 Société Malienne pour la Gestion de l’Eau Potable (SOMAGEP), BP E 708 Bamako, Mali; mamadou.issabre@somagep.ml; 8 Neogen Corporation, Lansing, MI 48912, USA; roztung@gmail.com

**Keywords:** water quality, water testing, monitoring, presence-absence, Colitag™, urban water, sub-Saharan Africa

## Abstract

Screening for fecal contamination via microbial water quality monitoring is a critical component of safe drinking water provision and public health protection. Achieving adequate levels of microbial water quality testing, however, is a challenge in resource-limited settings. One strategy for addressing this challenge is to improve the efficiency of monitoring programs. In African countries, quantitative microbial testing methods are commonly used to monitor chlorinated piped water systems. However, presence/absence (P/A) tests may provide an appropriate alternative for water supplies that generally show negative fecal contamination results. This study compares 1048 water quality test results for samples collected from five African urban water systems. The operators of the systems conducted parallel tests on the 1048 samples using their standard quantitative methods (e.g., most probable number or membrane filtration) and the Colitag™ method in P/A format. Combined data demonstrates agreement rates of 97.9% (1024/1046) for detecting total coliforms and 97.8% (1025/1048) for detecting *E. coli.* We conclude that the P/A test offers advantages as a simpler and similarly sensitive measure of potential fecal contamination for large, urban chlorinated water systems. P/A tests may also offer a cost-effective alternative to quantitative methods, as they are quicker to perform and require less laboratory equipment.

## 1. Introduction

The ingestion of fecal pathogens transmitted by contaminated water, food, and hands are the primary bases of diarrhea, a leading cause of sickness and death among children under the age of five [1–4]. Increasing evidence suggests that exposure to fecal pathogens also contributes to malnutrition and childhood stunting, even in the absence of diarrhea [5–7]. Water quality improvements can reduce diarrheal disease levels [3,4,8], and microbial water quality testing is a critical component of water safety management [9].

In low-income countries, institutional responsibilities for regulated water quality monitoring are generally well-defined in national laws and regulations [10,11]. Urban water suppliers usually have legal responsibilities and established parameters for operational monitoring. However, capacity for operational monitoring varies greatly. Large utilities typically maintain fully equipped microbiological laboratories, while smaller water suppliers and surveillance agencies more commonly rely on testing kits, or in low-resource areas, completely lack testing equipment for water quality monitoring [11].

The diversity of fecally transmitted pathogens, which include viruses, bacteria, protozoa, and helminths, makes it impractical to monitor water safety by testing samples for the presence of specific pathogens. Instead, water suppliers and public health agencies rely on indicator species to assess microbial drinking water quality: *process indicators* monitor the efficacy of water treatment and the integrity of distribution networks; *fecal indicators* identify the presence of fecal contamination [12]. Most national regulations, which are often based on World Health Organization (WHO) guidelines, specify the broad group of coliform bacterial species (total coliforms) as process indicators. Fecal indicators generally include thermotolerant coliforms, a subset of the total coliform group, and *Escherichia coli (E. coli)* species, which are a subset of thermotolerant coliforms [12].

Sampling frequency requirements for water suppliers are generally established based on population served. For example, the United States Environmental Protection Agency (EPA) requires a minimum of 1200 samples per year for a water supplier serving a population of 100,000, while WHO guidelines recommend 12 samples per year for the same population [9,13]. In Sub-Saharan Africa (SSA), most institutions do not achieve testing levels specified by country standards or WHO’s Guidelines for Drinking Water Quality (GDWQ) [14]. Sampling programs could be improved by increasing sampling rates and specifying how to increase sampling after contamination has been detected [15].

Common methods for quantifying fecal indicator species in water samples include direct quantification of colony forming units (CFUs) via Membrane Filtration (MF) techniques and estimates of the Most Probable Number (MPN) of bacteria via broth-culture assays [16]. MF and MPN procedures, however, are laborious to both perform and analyze. Studies have also shown that simpler P/A methods, which utilize similar growth and detection media as the quantitative techniques, are equally sensitive in detecting fecal indicator species in water samples from drinking supply systems in the United States [17–21].

Consequently, the United States Environmental Protection Agency (U.S. EPA) issued the Total Coliform Rule (TCR) in 1989 [22], which specifies the use of P/A assays for total coliform contamination to evaluate microbial drinking water safety. The TCR was revised in 2013, with minor corrections in 2014. The Revised TCR (RTCR) established regulatory requirements based on the number of samples that test positive for the presence of total coliforms, i.e., the frequency-of-occurrence [13,23]. For example, a public piped water system must undergo a full assessment if it exceeds a specified frequency of total coliform-positive samples per month, based on population served. If a sample tests positive for total coliforms, operators must collect a set of repeat samples within 24 h and also test for the presence of *E. coli*. A positive *E. coli* sample that is preceded or followed by a total coliform positive sample as part of repeat sampling requires rapid state and public notification as well as an assessment and corrective action [13].

The World Health Organization’s (WHO’s) Guidelines for Drinking Water Quality (4th edition) also state that P/A methods are appropriate for monitoring chlorinated water supply systems when the majority of tests for fecal indicator organisms provide negative results [9]. Among these systems, more frequent testing using simple methods is preferable to less frequent quantitative testing due to the increased likelihood of detecting contamination events [9]. For example, a study in India found that the P/A method is an effective screening method to detect coliform contamination in less polluted water sources, such as groundwater or piped supplies [24].

Water suppliers operating chlorinated distribution systems in large African cities, however, continue to rely on MF and MPN methods for monitoring microbial water quality [14], though their data indicate that microbial contamination is rarely detected: our previous compilation of over 27,000 test results from piped water systems across Africa showed that fecal indicator bacteria were only detected in 4% of samples from water piped to plots and 2% of samples from water piped to public taps and standpipes [25].

Based on these findings, we hypothesized that P/A methods are appropriate alternatives to quantitative microbial water quality assays for chlorinated piped water supplies in Africa. To test this hypothesis, we compared P/A and quantitative test results for water samples collected in five African cities: Ouagadougou, Burkina Faso; Abidjan, Côte d’Ivoire; Nairobi, Kenya; Bamako, Mali; and Dakar, Senegal.

## 2. Materials and Methods

This study was implemented through a collaboration between The Aquaya Institute and urban water suppliers in five African countries: L’Office National de l’Eau et de l’Assainissement (ONEA) in Burkina Faso; Société de Distribution d’Eau de le Côte d’Ivoire (SODECI) in Côte d’Ivoire; Nairobi City Water and Sewerage Company (NCWSC) in Kenya; Société Malienne de Gestion de l’Eau Potable (SOMAGEP) in Mali; and Sénégalaise des Eaux (SDE) in Senegal. These water suppliers operate and monitor large, chlorinated piped distribution networks in varied geographic settings and were interested in evaluating P/A microbial testing for their operational monitoring needs. The water suppliers conducted parallel tests for total coliforms and *E. coli* on a total of 1048 water samples using their established quantitative methods and a P/A method. We have listed these suppliers as sites 1–5 in the results in order to ensure their confidentiality.

### 2.1. Sample Collection

Between June 2015 and August 2016, the five water suppliers participating in this study collected approximately 200 water samples from their distribution networks according to the locations, frequencies, and quality control procedures outlined in their routine water sampling plans. The samples were collected as volumes of at least 200 mL in sterile vessels to provide sufficient quantities for two parallel tests (quantitative and P/A) on 100 mL from the following sources: piped networks (700 samples), treatment plants (100 samples), treated water reservoirs (79 samples), untreated groundwater (27 samples), and untreated surface water (82 samples). The water suppliers also assayed 33 negative control and 28 positive (spiked) control samples, which are further described in Section 2.3. Along with the water sample source, sampling staff recorded information into a standardized data collection sheet that included characteristics on water conditions at the time of sample collection (i.e., turbidity and free chlorine residual), the sampling location, and the date of sample collection (see Supplementary Material).

### 2.2. Microbial Testing Methods

The Membrane Filtration (MF) technique is widely used for the enumeration of coliforms and fecal coliforms in drinking water. The method consists of filtering a measured water sample through a cellulose acetate membrane with 0.45-µm diameter pores. Bacteria are retained on the surface of the membrane, which is incubated on a growth medium that selectively promotes multiplication and colony formation by coliform species. Incubation at 37 ^◦^C allows for the growth of all coliforms and incubation at 44.5 ^◦^C selects for thermotolerant species. Some media also differentiate between total coliforms and *E. coli* colonies according to the colors that each group produces after metabolizing specific compounds in the growth medium. Four of the five water suppliers that collaborated in this study used MF as their quantitative testing method (Table 1).

**Table 1:** Quantitative methods for microbial water testing employed by the five African water suppliers that participated in this study. The total number of tests includes analysis of positive and negative control samples.

Site	Number ofConnections	Quantitative Method	Media	Incubation	QAQCSamples	Total Number of Tests ^1^
1	361,475 [28]	Membrane filtration	Chromocult Agar	24 h at 35–37 ^◦^C	16	214
2	473,347 [29]	Membrane filtration	Chromocult Agar	21 ± 3 h at 36 ± 2 ^◦^C	51	198
3	582,502 [30]	Most Probable Number	MacConkey Broth	48 h at 35 ± 0.5 ^◦^C for coliform detection and 24 h at 44 ± 0.25 ^◦^C for *E. coli*	50	205
4	210,730 [31]	Membrane filtration	COMPASS Ecc Agar, Bile Esculin Azide Agar, Lactose TTC Agar with Tergitol 7	24 h at 37 ^◦^C for coliform detection and 24 h at 44 ^◦^C for *E. coli*	33	220
5	666,547 [32]	Membrane filtration	COMPASS Ecc Agar, Bile Esculin Azide Agar, Lactose TTC Agar with Tergitol 7	24 h at 37 ^◦^C for coliform detection and 24 h at 44 ^◦^C for *E. coli*	20	211^1^
**TOTAL**					**170**	**1048**

QAQC = Quality Assurance and Quality Control (positive or negative controls). TTC = Thermotolerant Coliforms.

1 For Total Coliforms, the total number of tests = 1046; the total number for site 5 = 209.

The multiple test tube method is a commonly employed Most Probable Number (MPN) assay for coliform indicator species in drinking water [26]. The method consists of inoculating a series of tubes containing selective growth medium with different dilutions of the water sample, screening for gas production resulting from lactose fermentation (a characteristic of coliform species) and conducting confirmatory tests that also check for *E. coli* in the samples. Results of the multiple test tube method are reported as a MPN: the statistical estimate of the mean number of coliforms present in the sample, which is based on the number of tubes (and corresponding dilution levels) that were confirmed for coliform growth [26]. In this study, only Site 1 used the MPN method (Table 1).

Colitag^TM^ served as the P/A method and is approved by the U.S. EPA for the simultaneous detection of total coliforms and *E. coli* in drinking water for both P/A and MPN formats [27]. If coliforms are present in the sample, the enzyme β-galactosidase will hydrolyze the chromogenic indicator ortho-nitrophenyl-β-D-galactopyranoside (ONPG) to release a yellow-colored compound. If *E. coli* are present in the sample, the enzyme β-glucuronidase will hydrolyze the fluorogenic indicator 4- methylumbelliferyl-β-D-glucuronide (MUG) to release a compound that fluoresces when exposed to longwave ultraviolet light. Fluorescence differentiates *E. coli* from other coliforms.

### 2.3. Quality Control

Sterile deionized or distilled water samples were used as negative controls (blanks) in each round of water quality testing. Positive control samples were generated by spiking sterile water samples with verified cultures of *E. coli.* In some cases, study partners did not have access to *E. coli* control strains and utilized likely contaminated sources to confirm media reactions (e.g., untreated groundwater or untreated raw water).

### 2.4. Data Analysis

To calculate rates of agreement between the quantitative and P/A methods, we created 2 × 2 contingency tables for total coliforms and *E. coli* [33]. We also compared testing methods by examining the numbers (fractions) of positive and negative results using logistic regression and chi-square (X^2^) tests [20]. We also performed the analysis on data disaggregated by institution (see Appendix A).

## 3. Results and Discussion

**Figure 1:**
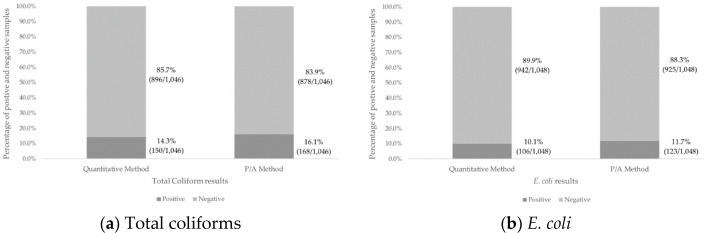
(a) Percentage of positive and negative samples for total coliforms according to P/A and quantitative diagnostic methods; (b) percentage of positive and negative samples for E. coli according to P/A and quantitative diagnostic methods. Due to the infrequent presence of fecal indicators in the distribution waters collected, naturally contaminated water sources were used to spike some of the samples; therefore, the percentage of positive results should not be used to draw conclusions about system water quality.

We conducted parallel tests on a total of 1048 water samples from the five urban water supply systems (Table 2). Comparisons of the proportions of samples that tested positive or negative for total coliforms and *E. coli* showed that the results of the two diagnostic methods were similar. Sixteen percent of the samples (168/1046) tested positive for total coliforms according to the P/A method and 14% (150/1046) tested positive for total coliforms according to quantitative methods. Twelve percent of the samples (123/1048) tested positive for *E. coli* according to the P/A method and 10% (106/1048) tested positive for *E. coli* using quantitative methods (Figure 1). There was no significant difference between testing methods (P/A vs. quantitative) when comparing fractions of positive samples for total coliforms (16% vs. 14%, *p* = 0.29) or *E. coli* (12% vs. 10%, *p* = 0.23) (Tables 3 and 4). Combined results from these tests demonstrated that the agreement rates between the quantitative and P/A methods were 97.9% (1024/1046) for total coliforms and 97.8% (1025/1048) for *E. coli* (Table 2). For the five samples with a negative P/A and positive quantitative result (two samples for total coliforms and three for *E. coli*), all had <5 CFUs for the quantitative method.

**Table 2:** (a) The 2 × 2 contingency table for total coliform with frequencies of positive and negative results provided by the partners’ established quantitative methods and the presence/absence (P/A) method; (b) the 2 × 2 contingency table for *E*. *coli* with frequencies of positive and negative results provided by the partners’ established quantitative methods and the P/A method.

	**Quantitative Method**					**Quantitative Method**		
	+	−	Total			+	−	Total
**P/A Method**	+ 148 − 2	20 876	168 878	**P/A Method**	+ −	103 3	20 922	123 925
	Total 150	896	1046		Total	106	942	1048
	(**a**) Total Coliforms					(**b**) *E. coli*		

**Table 3:** Comparison of total coliform detection between quantitative and P/A methods.

Number of Samples					% Positive				
Site	Total	Both Negative	Number Quantitative Positive	P/APositive	BothPositive	Quantit-ative	P/A	X^2^	p-Value
1	214	184	27	29	26	12.6%	13.5%	0.08	0.77
2	198	159	31	39	31	15.7%	19.8%	1.11	0.29
3	205	163	42	42	42	20.5%	20.5%	0.00	1.00
4	220	196	24	23	23	10.9%	10.5%	0.02	0.88
5	209	174	26	35	26	12.4%	16.8%	1.55	0.21
TOTAL	1046	876	150	168	148	14.3%	16.1%	1.20	0.27

**Table 4:** Comparison of *E. coli* detection between quantitative and P/A methods.

Number of Samples					% Positive				
Site	Total	Both Negative	Number Quantitative Positive	P/APositive	BothPositive	Quantit-ative	P/A	X^2^	p-Value
1	214	198	16	16	16	7.4%	7.4%	0.00	1.00
2	198	159	21	36	18	10.7%	18.3%	4.61	0.03
3	205	171	34	34	34	16.6%	16.6%	0.00	1.00
4	220	204	16	16	16	7.3%	7.3%	0.00	1.00
5	211	190	19	21	19	9.0%	10.0%	0.11	0.74
TOTAL	1048	922	106	123	103	10.1%	11.7%	1.42	0.23

We also disaggregated samples by institution (Tables 3 and 4). For total coliforms, there was no significant difference among testing methods when comparing the fractions of positive samples at the institution level (all *p* > 0.05) (Table 3). However, at one institution (Site 2) there was a significant difference between the two methods in *E. coli* detection—11% of samples tested positive for *E. coli* according to quantitative assays and 18% of samples tested positive according to the P/A method (*p* = 0.03) (Table 4).

The higher fraction of positive results for *E. coli* that were generated by the P/A method at Site 2 merits further discussion. While it is possible that some of these are false positives, the P/A method demonstrated a low false positive rate of only 1% for *E. coli* and 5.3% for total coliforms at 24 h in the U.S. EPA Alternative Test Procedure (ATP) approval study [34]. Among Site 2 samples that tested positive for *E. coli* using the P/A method and negative using the quantitative method, 5/18 were either positive control strains or raw water, which were likely to be true positives. The remaining samples were piped water samples, which likely contained chlorine-injured organisms. The P/A method was specifically designed to improve the detection of fecal contamination by resuscitating chlorine-damaged bacteria, which may have contributed to the higher rate of *E. coli* detection by the P/A method at Site 2 [35]. Site 1 samples, which used the same quantitative method as Site 2, however, did not reveal a significant difference in *E. coli* or total coliform recovery. These data suggest that the P/A method may be more accurate at detecting *E. coli* contamination for certain water sample types than the quantitative method, particularly when injured organisms are present. One study found that recovery performance of some methods may be affected by sample matrix differences, such as heterotrophic background growth, alkalinity, and pH, though Colitag™ produced high recovery rates regardless of sample type [36].

Furthermore, water testing results from the distribution network samples indicate low levels of contamination in the piped systems. According to the quantitative results, 96.3% (674/700) of distribution network samples tested negative for total coliforms and 99.1% (694/700) tested negative for *E. coli*. These low levels of contamination are comparable to our previous analysis of over 27,000 piped water samples in Africa, which found that 97% were free from fecal contamination [25]. Low levels of contamination are suitable for P/A tests when screening for fecal indicator bacteria.

The strong correlations between the results of the P/A and quantitative assays for microbial water quality indicators and the low levels of contamination in the piped distribution networks indicate that simple P/A tests, whose performance has been approved by an independent agency, such as the U.S. EPA, are appropriate alternatives to laborious quantitative diagnostics for monitoring well-managed chlorinated piped distribution networks in Africa. As most water quality monitoring institutions in sub-Saharan Africa do not achieve testing levels specified in standards or guidelines, the simpler P/A test may, therefore, provide a solution to increasing sampling rates. African water sector authorities should consider these results in their designs of water quality monitoring strategies and regulations.

However, even with regulatory acceptance of P/A methods, their application by African water suppliers and surveillance agencies will likely depend on the costs of procuring validated P/A tests that are imported from Europe or the United States (however, it is worth noting that most quantitative supplies are also imported from North America and the U.S.). The U.S. EPA considered reduced costs for P/A testing in comparison to quantitative methods when it adopted the Total Coliform Rule in 1989 [22]. Our previous analysis of reported testing costs in sub-Saharan Africa (including equipment, consumables, and labor) found that the per-test cost of H_2_S presence/absence tests (8.3 USD) was lower than the average per-test cost for membrane filtration (12.5 ± 8.1 USD) and MPN (14.0 ± 12.4 USD) [37]. However, this was not the case when only examining consumable costs: H_2_S presence/absence consumable costs (5.5 USD) were higher than average consumable costs for membrane filtration (3.1 ± 1.8 USD) or MPN methods (4.3 ± 3.9 USD) [37] (it is important to note that H_2_S cost data was only from one institution and no data was available on *E. coli* P/A methods). Similarly, a study by Bain et al. [16] that obtained costs from websites, catalogues, and quotations from manufacturers and suppliers found that the cost per test for P/A testing was approximately equal to quantitative methods (ranging from 0.6–5.0 USD and 0.5–7.5 USD, respectively, though these did not include import, delivery, or distributer mark-up prices in Africa). However, both studies found that equipment costs were generally lower for P/A compared to quantitative methods [16,37]. Furthermore, there is a possibility for additional cost savings from reduced staff time required for P/A methods (<5 min for sample handling) compared to quantitative methods (up to 30 min) [16].

A limitation of our study is that we did not generate all of our positive control samples using known control strains. Instead, in cases when water suppliers did not have access to control strains, they drew samples from likely contaminated sources, such as untreated groundwater or untreated raw water. Additionally, we were unable to conduct confirmatory tests of the positive results because of the capacity of the site laboratories.

The economic perspective outlined above suggests that despite the performance of the P/A test in monitoring chlorinated, piped distribution networks in African cities, the low consumable costs of quantitative methods will continue to favor their use over internationally validated, ready-to-use P/A tests that have higher per-unit costs, particularly among monitoring agencies that have already invested in the equipment and staff needed to perform quantitative methods. Nevertheless, validated P/A tests may still prove cost-effective in settings with chlorinated water supplies that are not supported by established laboratories.

## Appendix

**Table A1:** 2 contingency table for total coliform with frequencies of positive and negative results provided by Site 1 s established quantitative methods and the P/A method; (b) 2 × 2 contingency table for *E*. *coli* with frequencies of positive and negative results provided by Site 1^0^s established quantitative methods and the P/A method.

	Quantitative Method					Quantitative Method		
	+	−	Total			+	−	Total
**P/A Method**	+ 26 − 1	3 184	29 185	**P/A Method**	+ −	16 0	0 198	16 198
	Total 27	187	214		Total	16	198	214
	(**a**) Total Coliforms					(**b**) *E. coli*		

**Table A2:** 2 contingency table for total coliform with frequencies of positive and negative results provided by Site 2 s established quantitative methods and the P/A method; (b) 2 × 2 contingency table for *E*. *coli* with frequencies of positive and negative results provided by Site 2^0^s established quantitative methods and the P/A method.

	Quantitative Method					Quantitative Method		
	+	−	Total			+	−	Total
P/A Method	+ 31 − 0	8 159	39 159	P/A Method	+ −	18 3	18 159	36 162
	Total 31	167	198		Total	21	177	198
	(a) Total Coliforms					(b) *E. coli*		

**Table A3:** 2 contingency table for total coliform with frequencies of positive and negative results provided by Site 3 s established quantitative method and the P/A method; (b) 2 × 2 contingency table for *E*. *coli* with frequencies of positive and negative results provided by Site 3^0^s established quantitative method and the P/A method.

	Quantitative Method					Quantitative Method		
	+	−	Total			+	−	Total
P/A Method	+ 42 − 0	0 163	42 163	P/A Method	+ −	34 0	0 171	34 171
	Total 42	163	205		Total	34	171	205
	(a) Total Coliforms					(b) *E. coli*		

**Table A4:** 2 contingency table for total coliform with frequencies of positive and negative results provided by Site 4 s established quantitative methods and the P/A method; (b) 2 × 2 contingency table for *E*. *coli* with frequencies of positive and negative results provided by Site 4^0^s established quantitative methods and the P/A method.

	Quantitative Method					Quantitative Method		
	+	−	Total			+	−	Total
P/A Method	+ 23 − 1	0 196	23 197	P/A Method	+ −	16 0	0 204	16 204
	Total 24	196	220		Total	16	204	220
	(a) Total Coliforms					(b) *E. coli*		

**Table A5:** 2 contingency table for total coliform with frequencies of positive and negative results provided by Site 5 s established quantitative methods and the P/A method; (b) 2 × 2 contingency table for *E*. *coli* with frequencies of positive and negative results provided by Site 5^0^s established quantitative methods and the P/A method.

	Quantitative Method					Quantitative Method		
	+	−	Total			+	−	Total
P/A Method	+ 26 − 0	9 174	35 174	P/A Method	+ −	19 0	2 190	21 190
	Total 26	183	209		Total	19	192	211
	(a) Total Coliforms					(b) *E. coli*		

## References

[cit0001] Kotloff, K.; Blackwelder, W.; Nasrin, D.; Nataro, J.; Farag, T.; van Eijk, A.; Adegbola, R.; Alonso, P.; Breiman, R.; Faruque, A.; et al. The Global Enteric Multicenter Study (GEMS) of Diarrheal Disease in Infants and Young Children in Developing Countries: Epidemiologic and Clinical Methods of the Case/Control Study. Clin. Infect. Dis. 2012, 55, S232–S245. 23169936 10.1093/cid/cis753 PMC3502307

[cit0002] Liu, L.; Johnson, H.L.; Cousens, S.N.; Perin, J.; Scott, S.; Lawn, J.E.; Rudan, I.; Campbell, H.; Cibulskis, R.; Li, M.; et al. Global, Regional, and National Causes of Child Mortality: An Updated Systematic Analysis for 2010 with Time Trends since 2000. Lancet. 2012. 10.1016/S0140-6736(12)60560-1 22579125

[cit0003] Prüss-Ustün, A.; Bartram, J.; Clasen, T.; Colford, J.M.; Cumming, O.; Curtis, V.; Bonjour, S.; Dangour, A.D.; De France, J.; Fewtrell, L.; et al. Burden of Disease from Inadequate Water, Sanitation and Hygiene in Lowand Middle-Income Settings: A Retrospective Analysis of Data from 145 Countries. *Trop. Med. Int. Health* 2014, 19, 894–905. 24779548 10.1111/tmi.12329 PMC4255749

[cit0004] Wolf, J.; Hunter, P.; Freeman, M.; Cumming, O.; Clasen, T.; Bartram, J.; Higgins, J.; Johnston, R.; Medlicott, K.; Boisson, S.; et al. Impact of Drinking Water, Sanitation and Hand Washing with Soap on Childhood Diarrhoeal Disease: Updated Meta-Analysis and -Regression. Trop. Med. Int. Heal. 2018, 23, 508–525. 10.1111/tmi.13051 29537671

[cit0005] Humphrey, J. Child Undernutrition, Tropical Enteropathy, Toilets, and Handwashing. *Lancet* 2009, 374, 1032–1035. 10.1016/S0140-6736(09)60950-8 19766883

[cit0006] Lin, A.; Arnold, B.F.; Afreen, S.; Goto, R.; Huda, T.M.N.; Haque, R.; Raqib, R.; Unicomb, L.; Ahmed, T.; Colford, J.M.; et al. Household Environmental Conditions Are Associated with Enteropathy and Impaired Growth in Rural Bangladesh. Am. J. Trop. Med. Hyg. 2013, 89, 130–137. 23629931 10.4269/ajtmh.12-0629 PMC3748469

[cit0007] Ngure, F.M.; Reid, B.M.; Humphrey, J.H.; Mbuya, M.N.; Pelto, G.; Stoltzfus, R.J. Water, Sanitation, and Hygiene (WASH), Environmental Enteropathy, Nutrition, and Early Child Development: Making the Links. Ann. N. Y. Acad. Sci. 2014, 1308, 118–128. 24571214 10.1111/nyas.12330

[cit0008] Clasen, T.; Alexander, K.; Sinclair, D.; Boisson, S.; Peletz, R.; Chang, H.; Majorin, F.; Cairncross, S Interventions to Improve Water Quality for Preventing Diarrhoea (Review). Cochrane Database Syst. Rev. Interv. 2015 No. 10. 10.1002/14651858.CD004794.pub3 PMC4625648 26488938

[cit0009] WHO Guidelines for Drinking-Water Quality, 4th ed; World Health Organisation: Geneva, 2011.

[cit0010] Steynberg, M.C. Drinking Water Quality Assessment Practices: An International Perspective. Water Science and Technology. Water Supply 2002, 2, 43–49.

[cit0011] Rahman, Z.; Crocker, J.; Chang, K.; Khush, R.; Bartram, J A Comparative Assessment of Institutional Frameworks for Managing Drinking Water Quality. J. Water Sanit. Hyg. Dev. 2011, 1, 242–258.

[cit0012] Ashbolt, N.J.; Grabow, W.O.K.; Snozzi, M Indicators of Microbial Water Quality. In Water Quality: Guidelines, Standards, and Health; Fewtrell, L., Bartram, J., Eds.; IWA Publishing: London, UK, 2001; pp. 289–316.

[cit0013] U.S. EPA Revised Total Coliform Rule: A Quick Reference Guide; U.S. EPA: Washington, DC, USA, 2013.

[cit0014] Peletz, R.; Kumpel, E.; Bonham, M.; Rahman, Z.; Khush, R To What Extent Is Drinking Water Tested in Sub-Saharan Africa? A Comparative Analysis of Regulated Water Quality Monitoring. Int. J. Environ. Res. Public Health 2016, 13, 275. 10.3390/ijerph13030275 PMC4808938 26950135

[cit0015] Taylor, D.D.J.; Khush, R.; Peletz, R.; Kumpel, E. Efficacy of Microbial Sampling Recommendations and Practices in Sub-Saharan Africa. *Water Res.* 2018, 134, 115–125. 29407645 10.1016/j.watres.2018.01.054 PMC5842043

[cit0016] Bain, R.; Bartram, J.; Elliott, M.; Matthews, R.; McMahan, L.; Tung, R.; Chuang, P.; Gundry, S A Summary Catalogue of Microbial Drinking Water Tests for Low and Medium Resource Settings. Int. J. Environ. Res. Public Health 2012, 9, 1609–1625. 22754460 10.3390/ijerph9051609 PMC3386575

[cit0017] Bancroft, K.; Nelson, E.T.; Childers, G.W. Comparison of the Presence-Absence and Membrane Filter Techniques for Coliform Detection in Small, Nonchlorinated Water Distribution Systems. Appl. Environ. Microbiol. 1989, 55, 507–510. 2655537 10.1128/aem.55.2.507-510.1989 PMC184141

[cit0018] Clark, J.A. A Presence–Absence (P–A) Test Providing Sensitive and Inexpensive Detection of Coliforms, Fecal Coliforms, and Fecal Streptococci in Municipal Drinking Water Supplies. Can. J. Microbiol. 1968, 14, 13–18. 4868665 10.1139/m68-003

[cit0019] Jacobs, N.J.; Zeigler, W.L.; Reed, F.C.; Stukel, T.A.; Rice, E.W. Comparison of Membrane Filter, Multiple-Fermentation-Tube, and Presence-Absence Techniques for Detecting Total Coliforms in Small Community Water Systems. Appl. Environ. Microbiol. 1986, 51, 1007–1012. 3524452 10.1128/aem.51.5.1007-1012.1986 PMC239002

[cit0020] Pipes, W.; Minnigh, H.A.; Moyer, B.; Troy, M.A. Comparison of Clark’ s Presence-Absence Test and the Membrane Filter Method for Coliform Detection in Potable Water Samples. Appl. Environ. Microbiol. 1986, 52, 439–443. 3532953 10.1128/aem.52.3.439-443.1986 PMC203553

[cit0021] Rice, E.W.; Geldreich, E.E.; Read, E.J. The Presence-Absence Coliform Test for Monitoring Drinking Water Quality. *Public Health Rep.* 1989, 104, 54–58. PMC1580284 2493663

[cit0022] U.S. EPA National Primary Drinking Water Regulations: Total Coliform Rule. Fed. Regist. 1989, 54, 27543–27569.

[cit0023] U.S. EPA 40 CFR Parts 141 and 142: Revisions to the Total Coliform Rule; Final Rule; U.S. EPA: Washington, DC, USA, 2014.

[cit0024] Ramteke, P.W.; Pathak, S.P.; Bhattacherjee, J.W.; Gopal, K.; Mathur, N. Evaluation of the Presence-Absence (P-A) Test: A Simplified Bacteriological Test for Detecting Coliforms in Rural Drinking Water of India. Environ. mMnitoring Assess. 1994, 33, 53–59. 10.1007/BF00546661 24201701

[cit0025] Kumpel, E.; Peletz, R.; Mateyo, B.; Khush, R. Assessing Drinking Water Quality and Water Safety Management in Sub-Saharan Africa Using Regulated Monitoring Data. Environ. Sci. Technol. 2016. 10.1021/acs.est.6b02707 27559754

[cit0026] Bartram, J.; Pedley, S Microbiological Analyses. In Water Quality Monitoring—A Practical Guide to the Design and Implementation of Freshwater Quality Studies and Monitoring Programmes; Bartram, J., Ballance, R., Eds.; UNEP/WHO: Geneva, Switzerland, 1996.

[cit0027] U.S. EPA Expedited Approval of Alternative Test Procedures for the Analysis of Contaminants under the Safe Drinking Water Act; Analysis and Sampling Procedures; U.S. EPA: Washington, DC, USA, 2009; Volume 74, p. 57913.

[cit0028] Office National de l’eau et de l’assainissement (ONEA) Chiffres Clés: Indicateurs de Performance du Secteur en Milieu Urbain. Available online: http://oneabf.com/chiffres-cles/ (accessed on 7 February 2019).

[cit0029] Société de Distribution d’eau de la Côte d’Ivoire (SODECI) Données d’activité. Available online: http: //www.sodeci.ci/qui-sommes-nous/donnees-d-activite (accessed on 7 February 2019).

[cit0030] Water Services Regulatory Board A Performance Report of Kenya’s Water Services Sector—2015/16 and 2016/17. Available online: https://wasreb.go.ke/downloads/WASREB_IMPACT_Issue10_FINAL.pdf (accessed on 7 February 2019).

[cit0031] Société Malienne de Gestion de l’Eau Potable (SOMAGEP) SOMAGEP en Chiffres. Available online: http://www.somagep.ml/index.php/qui-sommes-nous/somagep-en-chiffres (accessed on 7 February 2019).

[cit0032] Sénégalaise des Eaux Magazine d’informations de la Sénégalaise des Eaux. Available online: http://www. sde.sn/Tlchargements/SDE_info-N$^\circ$37-Septembre2016.pdf (accessed on 7 February 2019).

[cit0033] Shaikh, S.A Measures Derived from a 2 × 2 Table for an Accuracy of a Diagnostic Test. J. Biom. Biostat. 2011, 02, 2–5.

[cit0034] CPI International US EPA ATP Study Report of Modified Colitag ATP Case No. D05-0035; CPI International: Santa Rosa, CA, USA, 2009.

[cit0035] CPI International Colitag: An Indole- and MUG-Based Medium for Detecting and Recognizing Fecal Coliforms and Escherichia Coli. In Proceedings of the ASM General Meeting, Dallas, TX, USA, 5–9 5 1991.

[cit0036] Olstadt, J.; Schauer, J.; Standridge, J.; Kluender, S. A Comparison of Ten USEPA Approved Total Coliform/E. Coli Test. J. Water Health 2007, 5, 267–282. 17674575

[cit0037] Delaire, C.; Peletz, R.; Kumpel, E.; Kisiangani, J.; Bain, R.; Khush, R. How Much Will It Cost To Monitor Microbial Drinking Water Quality in Sub-Saharan Africa? Environ. Sci. Technol. 2017, 51, 5869–5878. 28459563 10.1021/acs.est.6b06442 PMC5463268

